# Keap1/Nrf2 pathway in kidney cancer: frequent methylation of KEAP1 gene promoter in clear renal cell carcinoma

**DOI:** 10.18632/oncotarget.14492

**Published:** 2017-01-04

**Authors:** Federico Pio Fabrizio, Manuela Costantini, Massimiliano Copetti, Annamaria la Torre, Angelo Sparaneo, Andrea Fontana, Luana Poeta, Michele Gallucci, Steno Sentinelli, Paolo Graziano, Paola Parente, Vincenzo Pompeo, Laura De Salvo, Giuseppe Simone, Rocco Papalia, Francesco Picardo, Teresa Balsamo, Gerardo Paolo Flammia, Domenico Trombetta, Angela Pantalone, Klaas Kok, Ferronika Paranita, Lucia Anna Muscarella, Vito Michele Fazio

**Affiliations:** ^1^ Laboratory of Oncology, IRCCS “Casa Sollievo della Sofferenza” Hospital, San Giovanni Rotondo, Italy; ^2^ Genetic and Clinic Pathology Unit, University Campus Bio-Medico of Rome, Rome, Italy; ^3^ Unit of Biostatistics, IRCCS “Casa Sollievo della Sofferenza” Hospital, San Giovanni Rotondo, Italy; ^4^ Department of Bioscience, Biotechnology and Biopharmaceutics, University of Bari, Bari, Italy; ^5^ Department of Urology, Regina Elena National Cancer Institute, Rome, Italy; ^6^ Department of Pathology, Regina Elena National Cancer Institute, Rome, Italy; ^7^ Unit of Pathology, IRCCS “Casa Sollievo della Sofferenza” Hospital, San Giovanni Rotondo, Italy; ^8^ UOC of Urology, Campus Bio-Medico University Hospital, Rome, Italy; ^9^ Department of Genetics, University of Groningen, University Medical Center Groningen, The Netherlands

**Keywords:** ccRCC, KEAP1, methylation, epigenetic biomarker, outcome

## Abstract

The Keap1/Nrf2 pathway is a master regulator of the cellular redox state through the induction of several antioxidant defence genes implicated in chemotherapeutic drugs resistance of tumor cells. An increasing body of evidence supports a key role for Keap1/Nrf2 pathway in kidney diseases and renal cell carcinoma (RCC), but data concerning the molecular basis and the clinical effect of its deregulation remain incomplete.

Here we present a molecular profiling of the *KEAP1* and *NFE2L2* genes in five different Renal Cell Carcinoma histotypes by analysing 89 tumor/normal paired tissues (clear cell Renal Carcinoma, ccRCCs; Oncocytomas; Papillary Renal Cell Carcinoma Type 1, PRCC1; Papillary Renal Cell Carcinoma Type 2, PRCC2; and Chromophobe Cell Carcinoma).

A tumor-specific DNA methylation of the *KEAP1* gene promoter region was found as a specific feature of the ccRCC subtype (18/37, 48.6%) and a direct correlation with mRNA levels was confirmed by *in vitro* 5-azacytidine treatment. Analysis of an independent data set of 481 ccRCC and 265 PRCC tumors corroborates our results and multivariate analysis reveals a significant correlation among ccRCCs epigenetic *KEAP1* silencing and staging, grading and overall survival.

Our molecular results show for the the first time the epigenetic silencing of *KEAP1* promoter as the leading mechanism for modulation of *KEAP1* expression in ccRCCs and corroborate the driver role of Keap1/Nrf2 axis deregulation with potential new function as independent epigenetic prognostic marker in renal cell carcinoma.

## INTRODUCTION

Renal cell carcinoma is the most common malignant neoplasm arising in the kidney and comprises an heterogeneous group of tumors. Various histotypes of RCC have come to be defined on the basis of their histologic appearance, the presence of distinct driver mutations, varying clinical course, and different responses to therapy [1, 2]. On the basis of morphology RCCs are classified into clear cell carcinomas, papillary tumors, chromophobic tumors, oncocytomas, and collecting duct tumors. Clear cell Renal Cell Carcinoma (ccRCC) are the most common ones, with a frequency of 70–80% of all renal cancers. At time of diagnosis as many as one-third of the ccRCC patients may have a metastatic disease and about half of the patients will have a recurrence.

Papillary RCC, which accounts for 15% of RCCs encompasses type I papillary RCC (PRCC1) and type II papillary RCC (PRCC2). Although inhibition of the specific cellular signaling pathways has led to some clinical benefits, the effect is marginal and the prognosis remains poor for patients with advanced disease.

The premise that RCC histotypes might represent distinct diseases is underscored by multilevel genomics-based taxonomy studies. Moreover, different DNA methylation patterns have been characterized both across and within the histology-based subgroups, revealing different RCC molecular subtypes, some of these in association with a more aggressive behaviour [3].

In both clear cell and papillary histotypes the Keap1/Nrf2 pathway, the KEAP1 (Kelch-like erythroid-derived Cap-n-Collar Homology (ECH)-associated protein-1, *NFE2L2* (Nuclear factor (erythroid-derived 2)-like2, and *CUL3* (Cullin 3) were recently identified as probable drivers. This finding was consistent with the current knowledge that RCC belongs to the type of tumors in which the Nrf2 pathway was shown to be constitutively activated mainly by the loss of Keap1 functions that lead to Nrf2 nuclear accumulation and enhances the transcription of Phase II enzymes [4]. The induced activation of metabolizing enzymes confers to neoplastic cells resistance to radio- and chemotherapies with growth and survival advantages during their transformation and progression [5–6]. Moreover, the transcription factor Nrf2 plays an important role from acute kidney injury to chronic kidney disease and cancer and transcriptional activity of Nrf2 has been inversely correlated with FH enzyme activity, which is loss in PRCC2 [7–8].

Genetic alterations of Keap1/Nrf2 axis were described with a variable incidence in RCC, more frequently in PRCC2 [3, 9]. Genetic alterations of the Keap1/Nrf2 pathway were reported in a very small fraction of ccRCC patients. However, several studies demonstrated a general high impact of Nrf2 dysfunction in renal cell carcinoma, suggesting that the deregulation of the Keap1 may play a role in carcinogenesis process histotypes beside the presence of genomic alterations [10, 11]. We have previously reported that epigenetic modification by promoter methylation is a main mechanism of regulation of *KEAP1* gene expression in Non Small Cell Lung Cancer, malignant gliomas, breast cancer and that it was associated with worst progression free survival [12–16].

Since the role of DNA methylation and genes epigenetically altered in RCC have been an active area of research over the past decade, the main purpose of this study is to investigate the contribution of epigenetic deregulation of the *KEAP1* gene in different histotypes of renal cell carcinomas.

To address this issue, the analyses were stratified for the main five histological subtypes of renal cancer: 37 ccRCCs, 15 PRCC1s, 13 PRCC2s, 14 Oncocytomas and 13 Chromophobe Renal Cancers.

A clear association of *KEAP1* promoter methylation and the ccRCC histology was found in a training set of 37 cases with an incidence of 49%. A direct effect on Keap1 mRNA levels was demonstrated by *in vitro* experiments on a set of four ccRCC cell lines. The specific correlation between the *KEAP1* methylation and the Clear Cell histology was also validated by using two independent datasets of 481 ccRCC and 264 PRCC affected patients from *The Cancer Genome Atlas* (TCGA) portal, showing a significant correlation with the ccRCCs’ staging, grading and overall survival.

DNA-based assays are often more robust than RNA-based assays, and genes inactivated by promoter hypermethylation may provide a better target for molecular screening strategies to identify targeted therapies. Since the histologic appearance is considered the primary determinant in the classification of Renal Cancer [17], the discovery of specific variations in methylation profiles could further help to stratify the clear cell renal carcinoma subtype from others [3]. Moreover, our findings of *KEAP1* hypermethylation provide the first indication that this epigenetic mechanism is important also in the regulation of KEAP1 expression in an aggressive renal cancer histotype and could represents an additional and attractive diagnostic and prognostic biomarker.

## RESULTS

### Patients and treatment

Patients’ clinico-pathological features of all the Renal Cell Carcinoma histotypes analyzed into the study are summarized in Table 1. Overall, the series included 89 patients grouped in: 37 ccRCCs (41.6%), 15 PRCC1s (16.9%), 13 PRCC2s (11.2), 10 Chromophobe Renal Cell Carcinomas (14.6%) and 14 Oncocytomas (15.7%), respectively. The mean patient's age at the time of diagnosis was 63.0 ± 13.5 (yy ± SD) with a range from 23 to 86 years. More than half of the patients were men (68.6%) versus 31.4% of women. All patients underwent curative surgery and all malignant lesions were subjected to staging according to the TNM system (2009 classification): we found 38 T1 cases (50.7%), 13 T2 cases (17.3%), 21 T3 cases (28%) and 3 T4 cases (4%). According to the CAP guideline (College of American Pathologist) the Fuhrman grading was attributed only to ccRCC, showing a heterogeneous spectrum of aggressiveness: 1 case G1 (2.7%), 5 G2 (13.5%), 19 G3 (51.4%) and 12 G4 (32.4%).

**Table 1:** Clinical, tumor stages and histological features of RCC affected patients enrolled for the study (*n* = 89)

Characteristics	*n* (%)	ccRCC
**Age at the surgery (median (IQR))**	63 (23–86)	56 (48–70)
**Sex**	**89**	**37**
M	61 (68.6)	28 (76)
F	28 (31.4)	9 (24)
**Histology**	**89**	**37**
Clear cell	37 (41.6)	
Chromophobe	13 (14.6)	
Papillary tipe 1	15 (16.9)	
Papillary type 2	10 (11.2)	
Oncocytoma	14 (15.7)	
**Tumour Stage (excluded oncocytomas)**	**75 (84.2)**	
1a	14 (18,7)	4 (11)
1b	24 (32)	8 (22)
2a	9 (12)	
2b	4 (5.3)	10 (27)
3a	15 (20)	5 (13)
3b	6 (8)	3 (8)
4	3 (4)	0 (0)
**Lymph nodes stage (excluded oncocytomas)**	**75**	**30**
N0	20 (26.6)	16 (43)
N1	1 (1.4)	0 (0)
N2	7 (9.3)	4 (11)
Nx	47 (62.7)	17 (46)
**Clinical Metastasis stage**	**89**	**37**
M0	76 (85.3)	16 (43)
M1	13 (14.7)	21 (57)
**Fuhrman Grading (only ccRCC)**	**37**	
Grade I	1(2.7)	
Grade II	5 (13.5)	
Grade III	19 (51.4)	
Grade IV	12 (32.4)	
**PFS**	**89**	**37**
Yes	26 (29.2)	16 (43)
No	63 (70.8)	21 (57)
**OS (excluded oncocytomas)**	**75**	**37**
Death	7 (9.3)	3 (8)
Alive	68 (90.6)	34 (92)

Data are reported as median (IQR) for continuous variables and as frequencies and percentages for categorical variables; OS, Overall Survival.

Excluding benign lesions as oncocytomas, the lymph node involvement at the diagnosis was found only in 8 cases (N1 and N2 patients), whereas 67 patients didn't show lymphnode metastasis (Nx and N0 cases). During follow-up, 33 patients (37%) showed a relapse with 7 cases of local/ renal recurrence (7.8%) and 26 cases (29.2%) of distant metastasis (target organs: lungs, bone, liver and brain). The mean and median follow-up time was respectively 31.2 and 24.5 months with a range of 1–108 months.

During follow-up, 9 patients (10.1%) died of RCC, whereas 80 (89.9%) patients were still alive, with 92.2% survived at last follow-up. An Overall Survival equal to 90.6% was observed. Clinical characteristics of our series are in line with the just published data [18–19]. The number of cases for each histotype was chosen similar to perform an effective comparison. The number of ccRCCs was larger than the others since the inclusion of the two different metastatic and non-metastatic groups.

### *KEAP1* promoter methylation profile in renal carcinoma tissues

**Figure 1:**
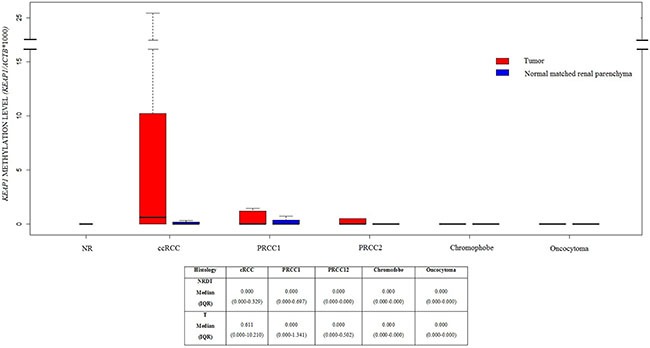
Boxplots of KEAP1 promoter methylation levels in Normal Renal tissues (NR), and different renal tumor histologies included into this study, paired with normal renal parenchyma distant from tumor Methylation levels are expressed as the (KEAP1/ACTB)*1,000. The boxes mark the interquartile range, (interval between the 25th and 75th percentile). ccRCC, clear cell Renal Cell Cancer; PRCC1, Papillary Renal Cell Carcinoma Type 1; PRCC2, Papillary Renal Cell Carcinoma Type 2.

The *KEAP1* methylation level was firstly evaluated on DNA obtained from a total of 89 RCC tissues, 70 paired normal renal tissues distant from the tumors (NRDT) and 10 normal renal tissues from patients affected by urothelial carcinoma (NR), (Supplementary Figures 2 and 3). The median values and Inter Quartile Ranges (IQR) for *KEAP1/ACTB* ratios were 0.0 (0–0) for NR, 0 (0.000–0.329), for NRDT paired with ccRCC, and 0.611 (0.000–10.210) for ccRCC tumor tissues, (Figure 1). The discriminatory power of the *KEAP1* QMSP assay was assessed by estimating the area under the ROC curve using paired normal renal tissues distant from tumor and ccRCCs. The AUC value was 0.68, with an optimal cut-off value of 1.56, a sensitivity of 49% and a specificity of 87%. In ccRCC methylation was detected in 18 out of 37 cases (48.6%), (Supplementary Figure 4). No statistically significant differences in methylation levels was detected between the NR and NRDT group. However, statistically significant differences were found when the NRDT and ccRCC group were compared (*p* = 0.0054; Wilcoxon Signed rank Test). No significant differences were found between NRDT and the other RCC histotypes.

**Figure 2:**
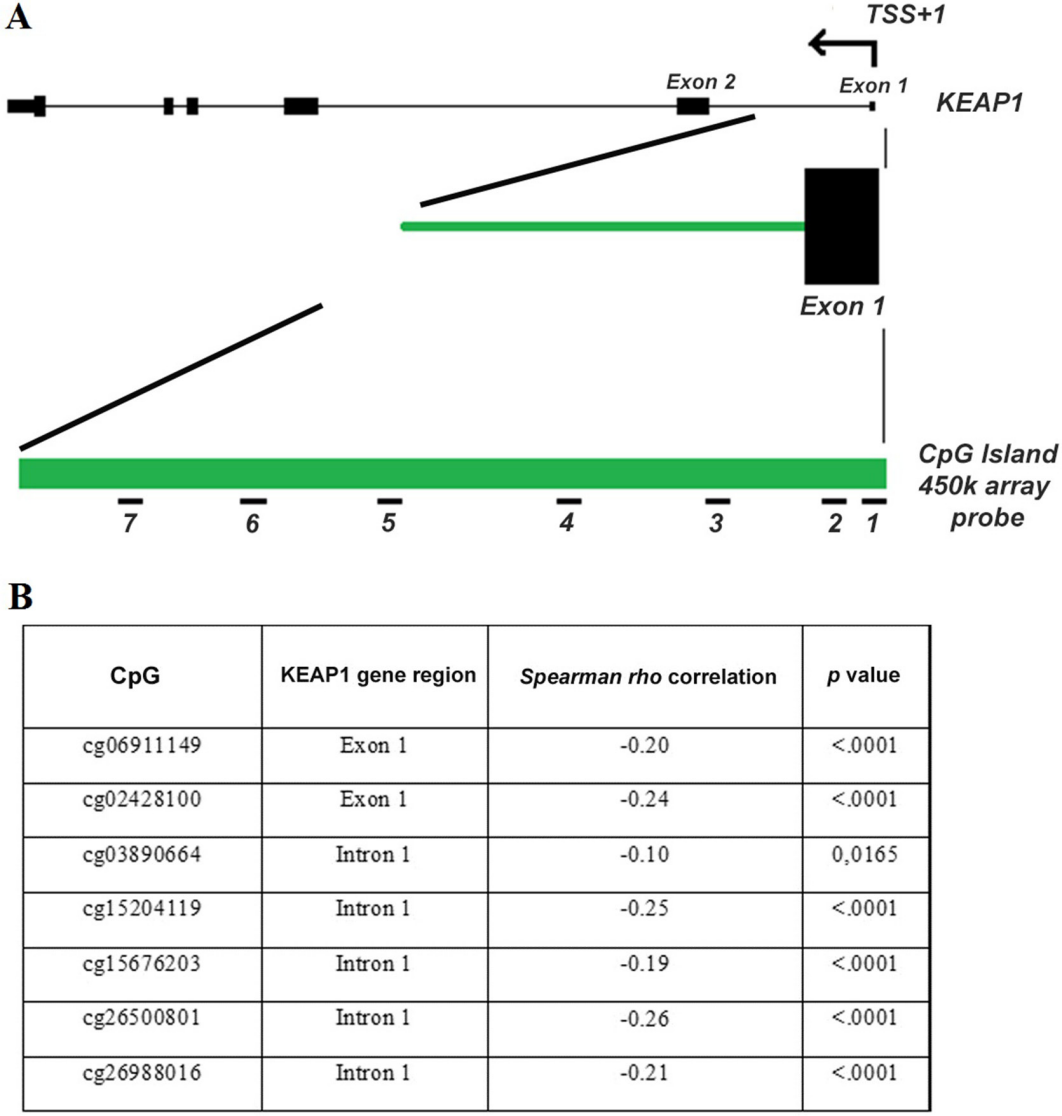
The 450 K methylation array data from the TCGA demonstrates that methylation of the KEAP1 promoter region is a hallmark in ccRCC (**A**) The KEAP1 gene structure, including its transcription start site (TSS), exons (black boxes) and introns (lines), a 1.2-kb CpG island of the KEAP1 gene (green bar) and the location of the probes present on the 450 K array (short black numbered lines, denoted as probes 1-7 for target ID cg06911149 and cg02428100 located into the exon 1 and cg03890664, cg15204119, cg15676203, cg26500801, cg26988016 located into the intron 1). (**B**) Inverse correlation of KEAP1 mRNA expression with the DNA methylation status in ccRCC. p: Spearman correlation coefficient.

In order to independently validate the specific correlation of data obtained to the ccRCC histotype, the *KEAP1* methylation and the functional effect of *KEAP1* promoter methylation on its expression was analyzed in two independent datasets of 481 ccRCC and 265 PRCCs cancer samples available from The Cancer Genome Atlas portal (TCGA), (for ccRCC cohort characteristics see additional Supplementary Table 2). The *KEAP1* gene has a 1.2 kb CpG island ( chr19:10613047-10614280, hg19/GRCh37) extending from the promoter region to intron 1, that is recognized by seven probes (denoted as 1 to 7) present on the Illumina Human-Methylation450 Bead Chip, all near the transcription start site of the *KEAP1* gene (Figure 2A).

A highly significant inverse correlation of aberrant *KEAP1* promoter methylation and *KEAP1* mRNA expression was found in both ccRCC and PRCC samples showing a methylation value > 0,10 (Figure 2B). However, a methylation value > 0,10 was observed only in 3% (7 out of 265) of PRCCs, thus confirming results obtained on our small cohort of an inconsistent contribution of the *KEAP1* promoter epigenetic silencing in this histology, (Supplementary Figure 5).

### Association of *KEAP*1 methylation with clinical parameters in ccRCCs

We finally examined the correlation between the *KEAP1* QMSP methylation level and patients’ clinical data. Specifically, we did not find any significant correlation between the *KEAP1* methylation level and lymph node status, or grading. Moreover, there was no significant association between *KEAP1* methylation and disease free survival, local recurrence or overall survival. Taking into account the few samples available, the same correlation analysis was then performed on the TCGA ccRCC dataset. For each CpG site of the *KEAP1* promoter gene region, the samples were stratified according *KEAP1* methylation median level to estimate the association with clinical parameters as independent prognostic molecular marker.

Relevant results were summarized in Table 2: CpG methylation at exon 1 and intron 1 revealed significant association with Overall Survival (OS), grading, staging and tumor dimension. No association was found with age and sex, lymphonodes count and metastasis.

**Table 2:** Prognostic association of CpGs located into the KEAP1 promoter region with clinical

CpG Island	*KEAP1* gene location	OS	Fuhrman Grade	Staging	T	KEAP1 expression (*r*, *p* value)
cg06911149	exon 1	0.01	0.0107	0.0005	0.0003	−0.20, <.0001
cg02428100	exon 1	0.426	0.0287	0.027	0.008	−0.24, <.0001
cg03890664	intron 1	0.139	0.9084	0.1388	0.2529	−0.10, 0,0165
cg15204119	intron 1	0.002	0.0019	< 00001	0.0015	−0.25, <.0001
cg15676203	intron 1	0.009	0.0037	0.0007	0.0005	−0.19, <.0001
cg26500801	intron 1	0.013	0.0549	0.0126	0.0183	−0.25, <.0001
cg26988016	intron 1	< 0.001	0.5512	0.0399	0.0147	−0.21, <.0001

characteristics and Overall Survival.

### Restoration of KEAP1 expression correlated with demethylation by 5-aza-dC treatment

**Figure 3:**
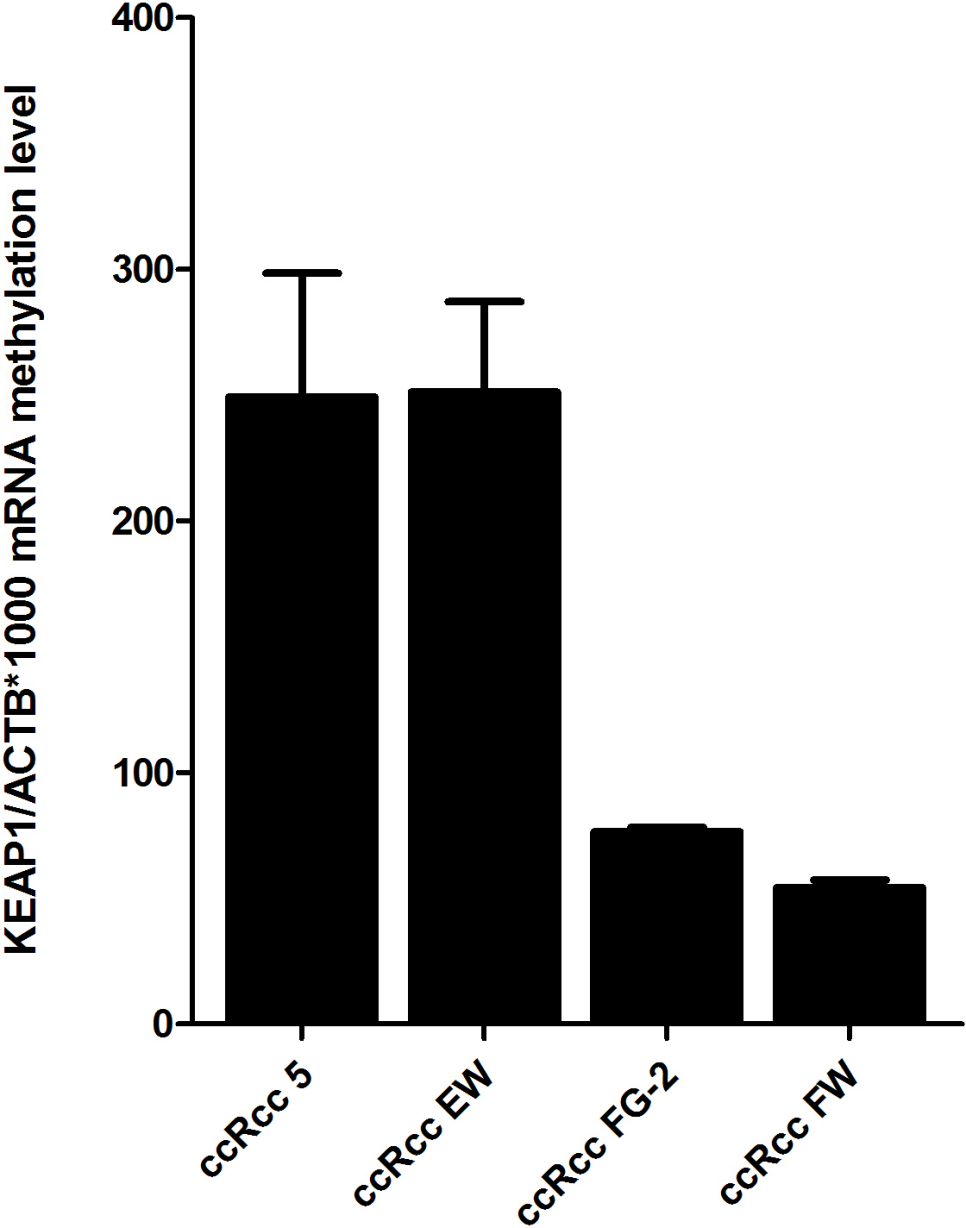
KEAP1 promoter methylation levels in four ccRCC cell lines (5, EW, FG-2, FW) detected by using quantitative methylation real-time PCR Values were reported as the KEAP1/ACTB ratio*1000. Error bars indicate the standard error of three different experiments.

**Figure 4:**
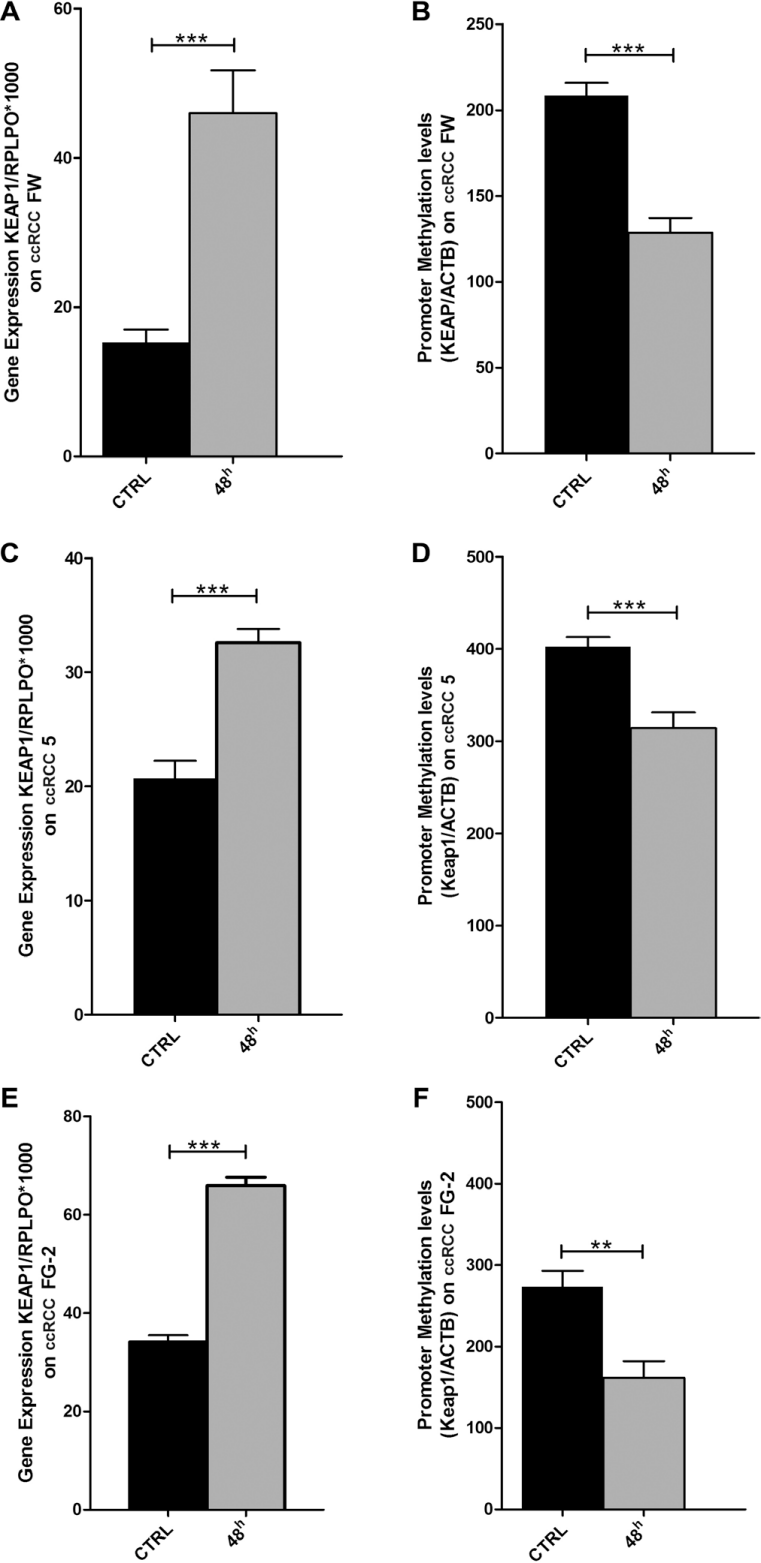
Changes in KEAP1 mRNA transcript levels in the (**A**) ccRCC-FW, (**C**) ccRCC 5, (**E**) ccRCC FG-2 cell lines by quantitative real-time RT-PCR before (CTRL) and after treatment with 5 μm of 5-azacytidine at 48 hours (AZA 48 h). Error bars indicate the standard deviation of three different experiments. Changes in KEAP1 promoter methylation levels in the (**B**) ccRCC FW, (**D**) ccRCC 5, (**F**) ccRCC FG-2 cell lines by quantitative methylation real-time PCR before (CTRL) and after treatment with 5 μm of 5-azacytidine at 48 hours (AZA 48 h). Error bars indicate the standard deviation of three different experiments. **p < 0.01, ***p < 0.001.

The methylation status of the four *ccRCC cell lines FG-2, FW, 5 and EW* was assessed. QMSP analysis showed a variable methylation level of *KEAP1* (Figure 3). To verify whether the repression of *KEAP1* expression was correlated with CpG methylation in the gene promoter, we examined the variation of *KEAP1* mRNA level in the four cell lines before and during treatment with 5-aza-dC (Figure 4A, 4C, 4E). By real-time quantitative PCR analysis a progressive increase in the KEAP1 transcript abundance was observed after 48 h (*p* < 0.0001) and was shown to correlate in ccRCC FW, ccRCC FG-2, ccRCC 5 with a decreased *KEAP1* promoter methylation at 48 h (*p* < 0.001 respectively), (Figure 4B, 4D, 4F). Keap1 expression did not reveal any significant variation in the ccRCC EW.

### KEAP1 deregulation is not caused by somatic alterations in the DGR domain of *KEAP1* or Nhe2 domain of *NFE2L2* in renal cell carcinoma

An alternative mechanism to achieve the deregulation of KEAP1 would be the occurrence of somatic mutations in our RCC samples. To exclude this possibility we analysed 20 ccRCCs (11 *KEAP1* methylated and 9 unmethylated samples) and 10 PRCC2 tumors for somatic alterations in the most frequently mutated coding regions of the *KEAP1* and *NFE2L2* genes. Specifically, we search for molecular lesions in the *KEAP1* Double Glycin Region (DGR) domain, which contains binding sites for Nrf2, Bcl2, Pgam5 and Ikkβ proteins. We also check for somatic alterations in the Nhe2 domain of the *NFE2L2*. This analysis did not reveal any sequence variations that would likely result in a functional alteration in Keap1 or Nrf2 proteins, (Supplementary Table 2).

### *KEAP1* promoter methylation and Keap1 immunostaining

To assess the potential correlation between the Keap1 protein levels in ccRCCs and the epigenetic silencing of the *KEAP1* gene, 15 ccRCC tumor cases showing *KEAP1* promoter methylation and 14 tumors without methylation were analysed by immunohistochemical analysis.

**Figure 5:**
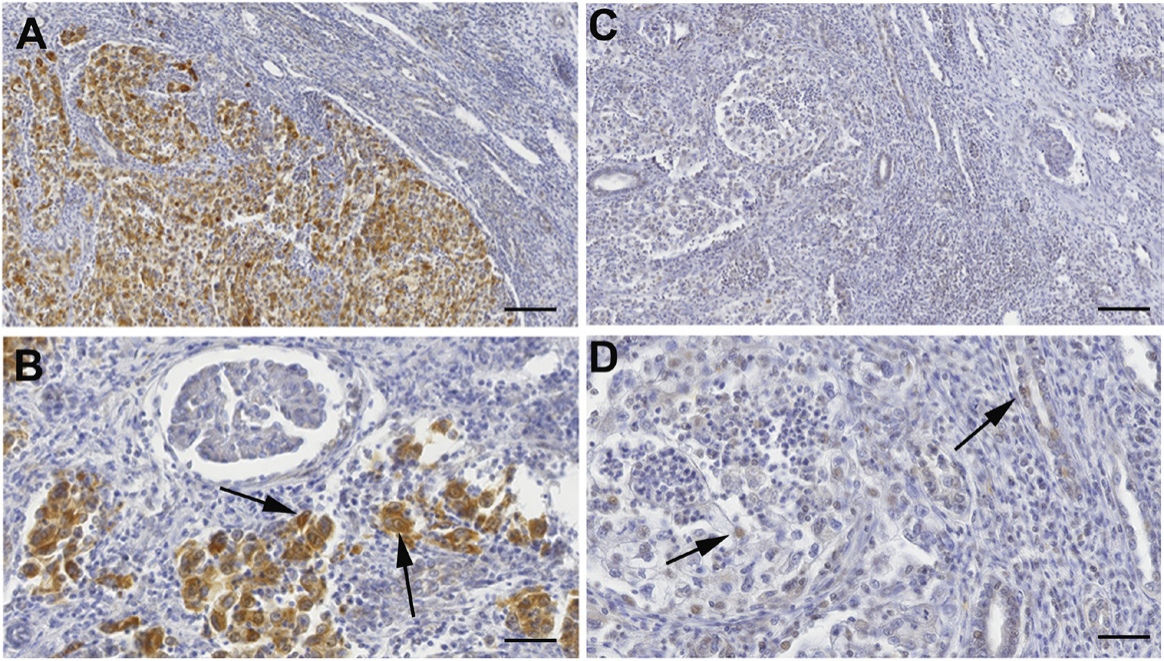
Keap1 protein expression by immunohistochemical analysis (**A**–**B**) Microphotographs are from a ccRCC sample negative for KEAP1 promoter methylation. (A) Original magnification X9. Scale bar 110 μm; (B) Original magnification X25. Scale bar 40 μm. Tissue-associated macrophages are positive for Keap1 staining and are used as internal positive controls. (**C**–**D**) Weak staining in one ccRCC case with KEAP1 promoter methylation. (C) Original magnification X9. Scale bar 110 μm; (D) Original magnification X25. Scale bar 40 μm. Tissue-associated macrophages are positive for Keap1 staining and are used as internal positive controls.

A semi-quantitative scoring system based on staining intensity and percentage of positive neoplastic cells to evaluate Keap1 immunoreactivity was applied. All the areas of the tissue section were evaluated for Keap1 protein expression. Cases were scored as positive if independently evaluated by two pathologists (PG and PP). Keap1 immunoreactivity was observed in the cytoplasm of tumor cells. Representative images of immunohistochemical staining are shown in Figure 5. A no statistically significant difference was observed.

## DISCUSSION

A growing amount of evidence suggest that an increased activity of Nrf2 due to *NFE2L2* or *KEAP1* mutations may play a pivotal role in the pathogenesis of many solid tumors and recently emerged as one of the main pathways implicated in Renal Carcinoma [13, 20–22]. Of note, deregulation of Keap1/Nrf2 pathway was highlighted in the Papillary Type 2 subtype of renal cancer as a consequence of an abnormal fumarate accumulation in congenital fumarate hydratase deficiency [13, 20] which predisposes to PRCC2.

Somatic mutations in *NFE2L2* and *CUL3* genes were also frequently reported in sporadic cases with PRCC2, but less frequently for the clear-cell subtypes of RCC. Somatic mutations in *NFE2L2* and *CUL3* genes were also reported more frequently for sporadic cases with PRCC2 than for the clear-cell subtypes of RCC [21, 23].

This could be explained taking into account the heterogeneity features of renal cancer and more importantly by an existing alternative epigenetic mechanism to regulate Keap1-Nrf2 signalling. *KEAP1* promoter epigenetic silencing as just reported in cancers as well as in the other diseases with a clear association with tumors having a low incidence of somatic mutations [22, 24].

Since no data are available regarding the contribution of *KEAP1* hypermethylation in renal cancer, we decided to investigate this epigenetic mechanism of silencing by performing a comprehensive genetic and epigenetic analysis in 89 surgical resected RCCs. As main result, an aberrant promoter *KEAP1* methylation was identified in the ccRCC subset with a frequency of 49%. The strong correlations with this specific histotype was then confirmed by the revision of the TCGA data for two different cohorts of 481 ccRCCs and 265 PRCCs. Moreover, we demonstrated that the methylation of the *KEAP1* CpG island represents a critical mechanism regulating the transcription levels of the *KEAP1* gene by showing a statistical significant inverse Pearson Correlation between *KEAP1* methylation and transcript levels. Further, these data are corroborated by the fact that pharmacological 5-Aza treatment is able to restore the expression of Keap1 in 3 out of 4 different ccRCC cell lines.

Methylation levels did not correlate with the Keap1 protein expression assessed in 29 ccRCC tumors by IHC analysis. The reasons for this lack of association might be due to several reasons. First, there is a lack of a consistently defined cut-off value for the semiquantitative immunohistochemical scoring and to detection limit [25]. Moreover, ccRCCs are heterogeneous/multifocal tumours and intra-tumoral heterogeneity could involve epigenetic changes similar to genetic and genomic events [26–27]. QMSP is a highly sensitive methodology and methylation signals may be obtained even if cells that carry KEAP1 promoter methylation represent a small proportion among the majority of cells with unmethylated promoter. Conversely, IHC may not be able to detect small clusters of cells that have lost protein expression.”

No genetic abnormalities were identified by analysing the DNA obtained from a subset of 20 ccRCCs and 14 PRCC2s. This result is in line with public data on the somatic mutation frequency of *KEAP1* in RCC. The COSMIC database has *KEAP1* sequence data on 953 cases of ccRCC of which only 8 had a non-synonymous *KEAP1* mutation (0.8%) and 1/875 had a non-synonymous *NFE2L2* mutation (0.1%). Moreover, in the TCGA analysed dataset, only 2 out of 446 ccRCCs had a mutations (0.5%). In papillary RCCs, 8 out of 183 non-synonymous *NFE2L2* mutations are described (4%), [28]. This result partially confirms the previous available published data that reported a low rate of inactivating mutations of *CUL3* or activating mutations of *NFE2L2* in sporadic PRCC2.

Deregulation of the Nrf2/Keap1 system has been linked to patient's outcome and to resistance to a variety of anticancer drugs, due to the key role of Nrf2 in modulating the expression of phase II drug metabolism enzymes and phase III drug transporters [17]. In patients affected by solid tumors, a nuclear Nrf2 accumulation in combination with a low or absent Keap1 expression was associated with poor outcome. However, this was not well investigated in Renal Cancer. In our study follow up data were available for all 37 ccRCC cases. No evidence of a statistical significant correlation with the risk to progress was observed for patients bearing epigenetic abnormalities. This may be mainly due to the statistical power of the small sample cohort. Therefore, we extended our studies by analysing 450K methylation array data for the CpG promoter of *KEAP1* for an independent cohort of 481 ccRCC patients with available clinic-pathological data. A clear association was observed between the hypermethylation of CpGs located in the *KEAP1* promoter and an increased ccRCC tumor grading and staging, further supporting their biological relevance. Furthermore, the data analysis suggests that *KEAP1* hypermethylation is able to predict patients’ survival. An increased methylation level of 5/7 CpGs was strongly associated with worse OS in ccRCCs.

Our study reports for the first time the epigenetic modulation of *KEAP1* by CpGs promoter hypermethylation as the leading mechanism of KEAP1 deregulation in ccRCC and together with recently published data on PRCC2, corroborate the hypothesis of a driver role of the Keap1/Nrf2 pathway in the ccRCCs subtypes with a specific epigenetic deregulation mechanism. In line with data from previous molecular studies, this epigenetic finding represents a new disease molecular marker for a specific subtype of RCC. An important avenue of future work will be to complement this epigenetic biomarker to existing renal cancer genetic biomarkers to assess their importance from the standpoint of therapy and clarify their potential for application in the clinical setting. At the same time, based on the high frequency of tumors displaying aberrant Nrf2 activation, Nrf2 should be regarded as an important new pharmacological target [29, 30].

## MATERIALS AND METHODS

### Cell lines

ccRCC FG-2, ccRCC FW, ccRCC 5 and ccRCC EW clear renal cancer cell lines were obtained in collaboration with the UMCG, Department of Genetics, Groningen, the Netherland (Dr. Klaas Kok, PhD) and were cultivated in RPMI 1640 medium, supplemented with 10% FBS. DNA was extracted from each cell line by using the standard procedure with Phenol-Chloroform. RNA was extracted using Trizol reagent (Life Technologies) in according to the manufacturer's instructions. DNA and RNA concentrations were estimated by NanoDrop Spectrophotometer ND-1000 (Thermo Scientific).

### Patients and tissue samples

The patients’ clinical and pathological data including pathological TNM staging, site of the lesion, grading, age, gender, and follow-up data were collected and are shown in Table 1.

Renal cancer tissues samples were obtained as Formalin Fixed Paraffin Embedded (FFPE) specimens from 89 patients surgically treated in the Department of Urology at the “Regina Elena” National Cancer Institute of Rome. All tissue specimens showing features of Clear Renal Cell Carcinoma (ccRCC, *n* = 37), Type I Papillary Renal Cell Carcinoma (PRCC1, *n* = 15), Type II Papillary Renal Cell Carcinoma (PRCC2, *n* = 13), Oncocytoma (*n* = 14) and Chromophobe Renal Cell Carcinoma (*n* = 13), attending the Outpatient Clinic of the Department of Urology or undergoing to curative surgical treatment during the period 2003–2013. Wherever possible, all available paired histologically confirmed normal renal tissues distant from tumor (NRDT, *n* = 70) and normal renal tissue samples from urothelial tumor patients (NR, *n* = 10) were collected. All human materials used in the study were obtained in compliance with guidelines of the Local Ethical Committee.

After excision, tissues were collected in 10% formalin and embedded in paraffin for histopathological and immunohistochemical analysis. Tumor samples with at least 70% cancer cells were eligible for direct genetic and epigenetic analyses. Cases showing tumor cellularity lower than 70% were microdissected for enrichment of neoplastic cell content.

### DNA extraction

Sections, 3-μm-thick, were cut from FFPE tissue blocks and subjected to Hematoxilyn and Eosin (H&E) staining to verify tumor cellularity. Under light microscope, 12-μm-thick sections of tumor specimens were then carefully dissected to enrich for areas that contained tumor cells. DNA was subsequently extracted from the paraffin-embedded sections as previously described [31]. DNA concentrations were measured by using Thermo Scientific NanoDrop^™^ 1000 Spectrophotometer (Thermo Scientific).

### Mutation analysis

DNA obtained from 20 ccRCC and 10 PRCC2 tissues was analysed by fluorescence-based direct sequencing of the entire *KEAP1* gene region encoding the DGR (Double Glycine Repeat) domain of the Keap1 protein and exon 2 of the *NFE2LE* gene, encoding the Nhe2 domain (Supplementary Table 3). Amplification reactions were performed by using the Gene Amp PCR System 9700 Thermal cycler (Applied Biosystem, Foster City, CA), in a final reaction volume of 25 μl containing 100 ng of genomic DNA template, 0.25 nM dNTPs, 20 pmol of each primers, 1U HotMaster Taq polymerase (Eppendorf AG, Hamburg, Germany), in 10X Hot Master Taq Buffer with magnesium and sterile distilled water. PCR cycling conditions include an initial denaturation step at 94°C for 2 min, followed by 35 cycles of 94°C for 30 sec, annealing for 30 sec, extension at 72°C for 30 sec and ending with a final elongation step at 72°C for 7 min. PCR products were purified using GFX PCR DNA and the Gel Band Purification Kit (GE Healthcare, Buckinghamshire, UK) and sequenced by using the Big Dye Terminator Ready Reaction mix v. 1.1 on an ABI 3100 sequence detection system with the Sequencing Analysis software v.3.7 (Applied Biosystems).

### Bisulfite conversion of DNA and quantitative methylation specific PCR analysis (QMSP)

One microgram of DNA extracted from cell lines and tissue samples was subjected to bisulfite treatment and DNA purification using the Epitect Bisulphite kit (Qiagen Sci, MD, USA) according to manufacturer's instruction. Bisulfite-modified DNA was used as template for Quantitative Methylation Specific PCR (qMS-PCR) to detect converted DNA. Primer/probe sets for the *KEAP1* promoter region and for the unmethylated promoter region of the *ACTB* as reference gene were previously described and are reported in Supplementary Table 1 [12]. Calibration curves for both target and reference genes were constructed using serial dilutions (90–0.009 ng) of commercially available fully methylated DNA (CpGenome Universal Methylated DNA, Millipore, Chemicon, cat#S7821). Amplification reactions were carried out in triplicate in 384-well plates and in a volume of 10 mL that contained 50 ng of bisulfite-modified DNA on a ABI PRISM 7900 Sequence detection system and were analysed by SDS 2.1.1 software (Thermo Fisher Inc., Applied Biosystems division). Each plate included calibration curves for the *ACTB* and *KEAP1* genes, patients’ DNA samples, a positive control CpGenome Universal Methylated DNA, and multiple water blanks (Supplementary Figure 1). The Cp (cross point) values of each QMSP reaction were calculated using the second derivative maximum method. The qMS-PCR standard curves of the *KEAP1* and *ACTB* genes for the normalization of the input DNA were established with CpGenome Universal Methylated DNA. Methylation levels were finally calculated as the ratio of *KEAP1* to *ACTB* and then multiplied by 1000 for easier tabulation (average value of triplicates of Target Gene/average value of triplicates of *ACTB*× 1000).

### *In vitro* 5-Aza-2′-deoxycytidine (5-aza-dC) treatment

FG-2, FW, 5 and EW ccRCC cell lines were seeded in a 6 well dish. The 5-aza-2′-deoxycytidine (DAC), an inhibitor of DNA methyltransferase, was added in a concentration of 5 μM (Sigma-Aldrich) with fresh medium for 24 h and 48 h. At both time points (24 h and 48 h) cells were harvested for DNA and RNA isolation to interrogate the effects of induced DNA demethylation and analyse the KEAP1 expression levels.

### Quantification of the KEAP1 expression by real-time PCR

PCR fragments for *KEAP1* and *RPLPO* were amplified by the Taqman assay listed in Supplementary Table 1, and were cloned into the StrataClone^TM^ PCR Cloning Vector pSC-A (Stratagene, Milan, Italy). Mini-prep cultures were grown in 5 ml of LB-Ampicillin broth. Plasmid DNA from the selected transformed cells was isolated using the QIAprep^®^ Spin Miniprep Kit (Qiagen) Five plasmid dilutions in the range of 1 × 10^6^ copies to 1 × 10^2^ copies were used to construct the standard curves for real-time PCR.

First strand cDNA synthesis from 1 μg of total RNA extracted from renal cell lines was carried out with SuperScript III First-Strand Synthesis (Thermo Fisher, Invitrogen Division, Carlsbad, CA, USA) using a gene expression amplification mixture containing 2.5 × TaqMan^®^ Universal PCR Master Mix (Thermo Fisher, Life Technologies division), 250 nM of TaqMan^™^ Gene Expression Assay with TaqMan probe and 1 μl of template cDNA or plasmid product (serial dilutions), (Table 1). Reactions were run on ABIPRISM 7900HT Sequence Detection System (Thermo Fisher, Life Technologies division). Protocol conditions were as follows: 10 min at 95°C, 40 cycles at 95°C for 15 s and 60°C for 60 s. Each assay was carried out in triplicate and the transcription level was normalized using *RPLPO* as reference gene. Calibration curves for the *KEAP1* and *PRLPO* genes (used as calculation method) were constructed and sample concentration was calculated using the plasmid standard curve, resulting in plasmid concentrations expressed as copy number of corresponding standard molecules. The relative sample amount was expressed as ratio marker ([*KEAP1/RPLPO*]*1000 for an easier tabulation).

### Immunohistochemistry (IHC)

Formalin fixed paraffin-embedded sections (3 μm) of 29 ccRCCs were selected for IHC analysis and collected on polarized slides. The sections were deparaffinised in xylene, hydrated in gradient alcohol, and warmed in Tris-EDTA buffer (0.01 M, pH = 9.0) for antigen retrieval at 98°C. The sections were then incubated with hydrogen peroxide (0.3% v/v) in methanol for 5 min to quench the endogenous peroxidase activity. Thereafter, the slides were incubated with rabbit polyclonal anti-Keap1 antibody (AP-20503, Proteintech, Chicago, USA) for 60 min at RT. The primary antibody was detected by using commercially available detection kit (EnVision^TM^FLEX+, Dako, Glostrup, Denmark) following the manufacturer's protocol and diaminobenzidine as chromogen.

Slides were washed with Tris-buffered saline (TBS, 0.1 M, pH = 7.4), 3–5 times after each step. Finally, the sections were counterstained with Mayer's hematoxylin and mounted with Biomount (BIO-OPTICA, Milan, Italy). In the negative control tissue sections, the primary antibody was replaced by isotype specific non-immune rabbit IgG. Tissue sections from colon cancer were used as a positive control for Keap1expression. The sections were evaluated by light microscopic examination on Olympus BX51 microscope.

Each slide was evaluated for Keap1 immunostaining by using a semi-quantitative scoring system combining staining intensity and the percentage of positive neoplastic cells.

Immunoreactivity was assessed in all the areas of the tissue section. Keap1 protein expression, independently evaluated by two Pathologists (PG and PP) was scored as positive if cytoplasm reactivity was observed in tumor cells. The semi-quantitative scale based on the % of immunoreactive neoplastic cells was: 0–10% = 0; 10–30% = 1; 30–50% = 2; 50–70% = 3 and 70–100% = 4. Sections were also scored on the basis of staining intensity as negative = 0; mild = 1; moderate = 2; intense =3. Finally, a immunoreactive staining score (IRS) was obtained by adding the score of percentage positivity and intensity and samples were classified ad negative/weak staining (IRS < 2) and moderated/strong staining (IRS ≥ 2).

### Validation of the *KEAP1* expression and promoter hypermethylation in two independent set of ccRCC and PRCC tumors (TCGA data analysis)

*KEAP1* Methylation and mRNA expression data for ccRCCs and PRCCs were downloaded from the “*The Cancer Genome Atlas*” (TCGA) data portal (https://tcga-data.nci.nih.gov). These data include *n* = 481 ccRCCs and *n* = 265 PRCC affected patients from two independent platforms: Illumina Infinium DNA methylation (HumanMethylation 450 K) and Illumina HiSeq gene expression arrays.

### Statistical data analysis

All analyses were performed using SAS Release 9.3 (SAS Institute, Cary, NC). A *p value* < 0.05 was considered for statistical significance.

Patients baseline characteristics were reported as mean and standard deviation or median and interquartile range and frequencies and percentages for continuous and categorical variables, respectively. Baseline comparisons were performed using Mann-Whitney *U*-test or Pearson chi-squared test for continuous and categorical variables, respectively.

Methylation level comparison between tumors and paired normal tissues was performed by using the Wilcoxon Signed Rank test. ROC curve analysis was performed to assess the diagnostic performances of *KEAP1* methylation levels. Sensitivity and specificity were also reported at the optimal cut-off which maximized jointly sensitivity and specificity.

Correlation between KEAP1 mRNA expression (TCGA Illumina HiSeq platform) and the level of *KEAP1* methylation was assessed using Spearman correlation coefficient.

Association between IHC and methylation was tested using Mc Nemar test.

Proportional hazard Cox regression analysis was performed to assess the association between *KEAP1* methylation and overall survival.

For *in vitro* experiments, group comparisons were performed using two-tailed Student's *t*-test.

## SUPPLEMENTARY MATERIALS FIGURES AND TABLES


