# Supplementary material for: Keap1/Nrf2 pathway in kidney cancer: frequent methylation of KEAP1 gene promoter in clear renal cell carcinoma
Source: Oncotarget. 2017 Jan 4;8(7):11187–98. doi: 10.18632/oncotarget.14492 (PMC5355256; doi:10.18632/oncotarget.14492)
Supplement: Supplementary file 1 [file oncotarget-08-11187-s001.pdf]

# Keap1/Nrf2 pathway in kidney cancer: frequent methylation of KEAP1 gene promoter in clear renal cell carcinoma

## Supplementary Materials

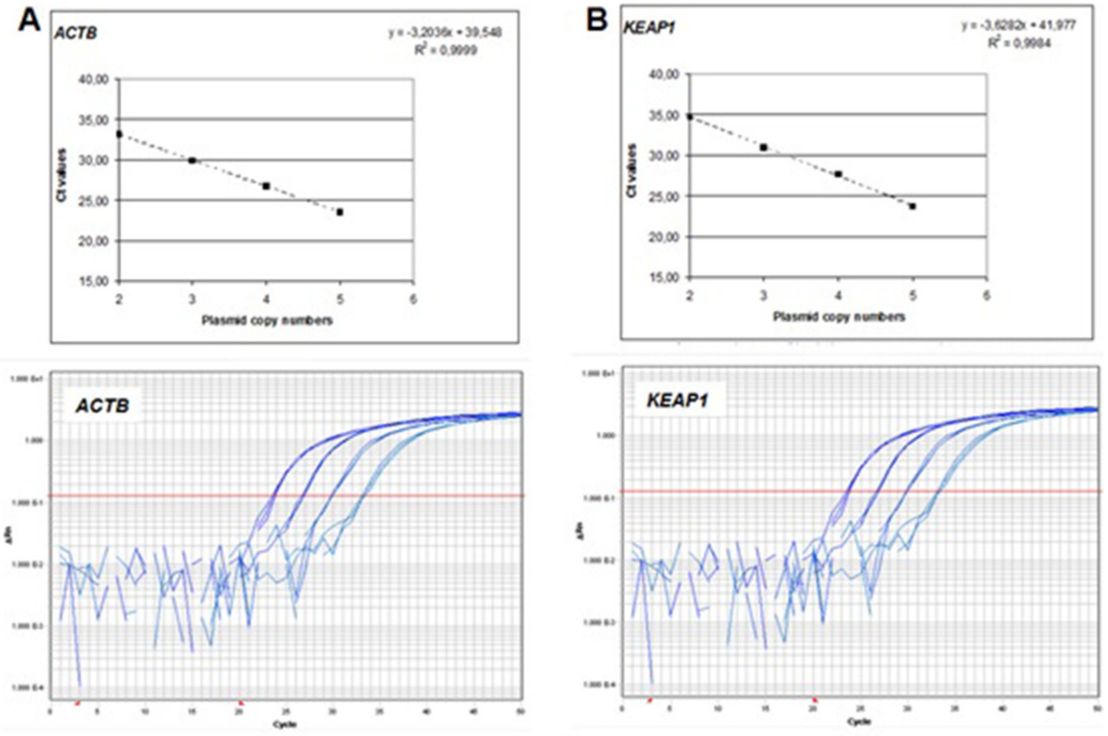


**Supplementary Figure 1:** Standard curves and amplification plot for *ACTB* (**A**) and *KEAP1* (**B**). The upper panels show the mean of Ct values versus log of plasmid copy numbers, measured in triplicate. Slope and intercept are represented in the equations of the regression lines, together with regression coefficient. The lower panels show the Ct values versus ΔRn.

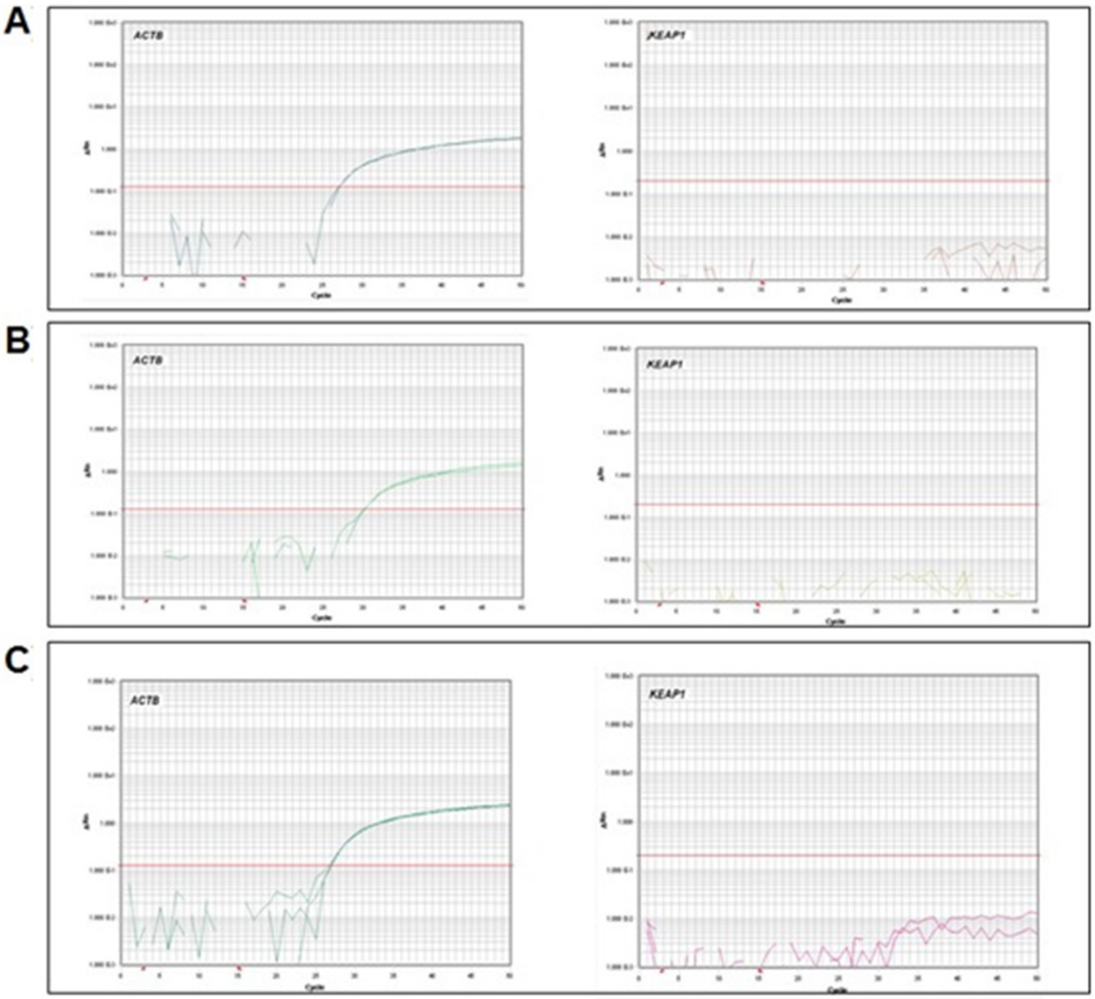


**Supplementary Figure 2:** Amplification plot for *ACTB* and *KEAP1* of three normal tissues without methylation at the *KEAP1* promoter region; (**A**) RCC-95 (**B**) RCC-98 and (**C**) RCC-101.

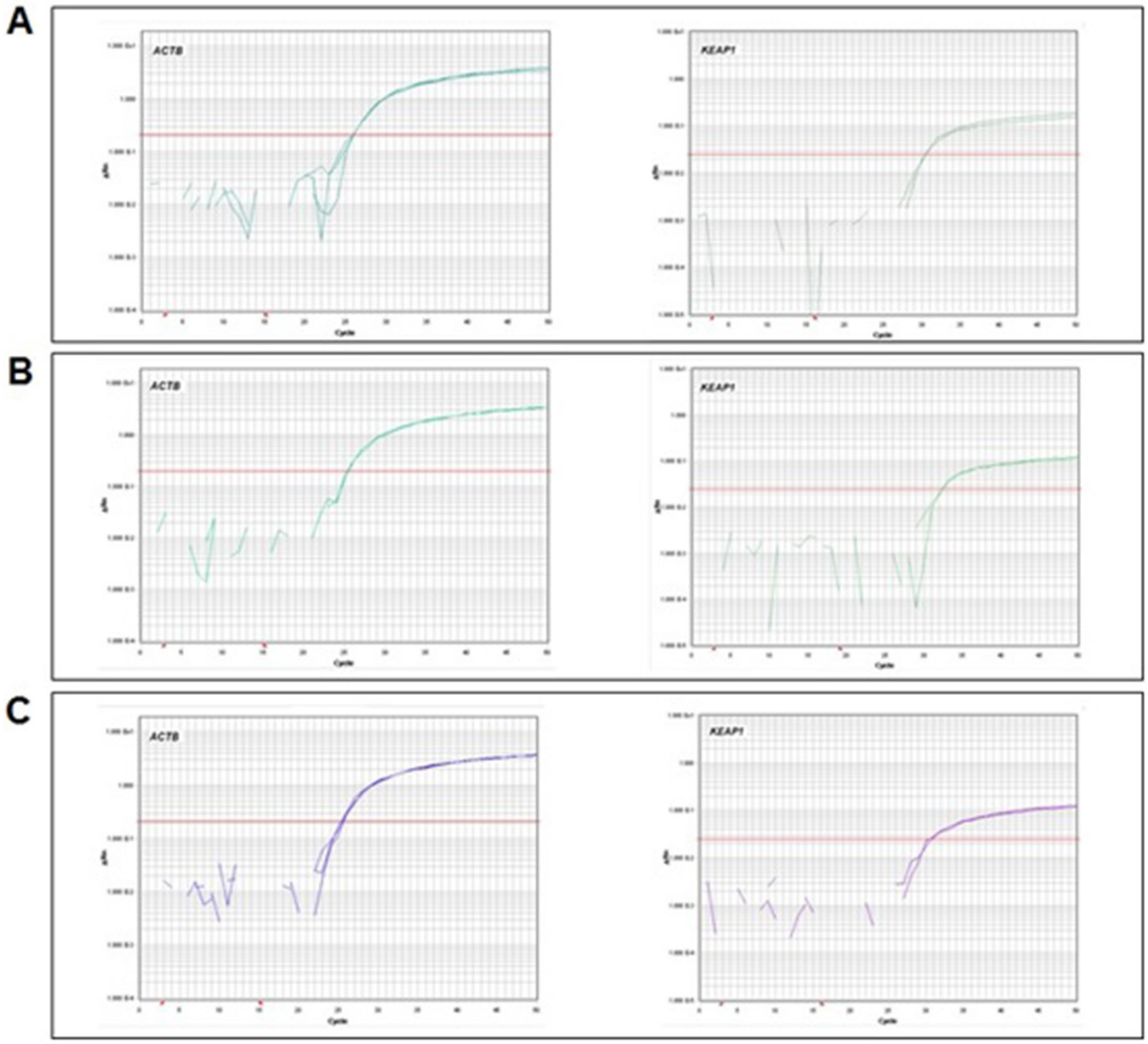


**Supplementary Figure 3:** Amplification plot for *ACTB* e *KEAP1* in three ccRCC tissues with *KEAP1* promoter methylation; (**A**) RCC-95 (**B**) RCC-98 and (**C**) RCC-101.

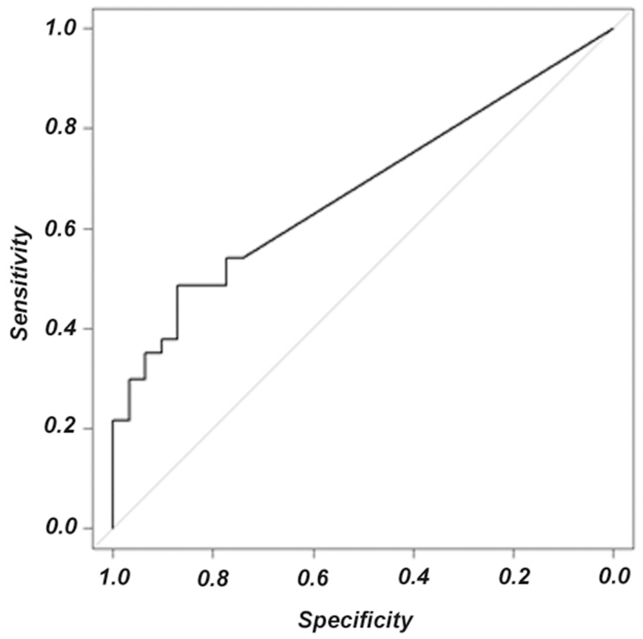


**Supplementary Figure 4: ROC curve analysis of Quantitative Methylation Specific PCR results.** The ROC curve for *KEAP1* QMSP assay was designed on the basis of *KEAP1/ACTB* ratios determined in normal renal tissues distant from tumor (NRDT) paired with ccRCCs.

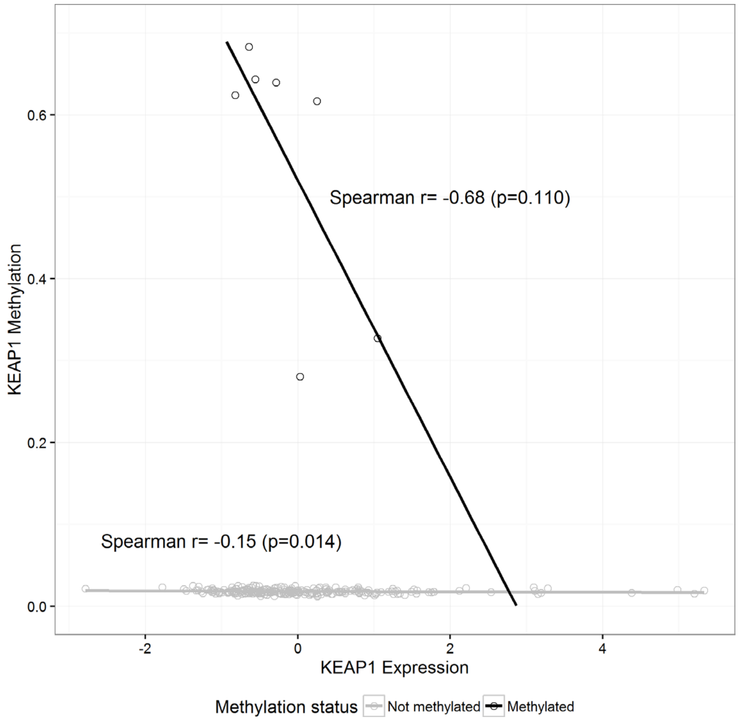


**Supplementary Figure 5: Scatterplot of KEAP1 mRNA expression vs. methylation level from TGCA data.** Two separated regression lines were estimated between methylated and unmethylated samples, defined using a methylation level cut-off of 0.10. Spearman correlation coefficients (r) were further reported.

**Supplementary Table 1: Primers sequence used for *KEAP1* and *NFE2L2* mutation screening and KEAP1 expression analysis**

| Primer name | primer sequence (5′ → 3′) | Annealing Temperature (°C) |
|---|---|---|
| **Screening Mutations** | | |
| KEAP1-Ex-3aF | TTGCAAAACGAGGCCCCGGC | 60 |
| KEAP1-Ex-3aR | TGCACTCAGTGGAGGCGTAC | |
| KEAP1-Ex-3bF | CTGCAGTCACAGTGCCCTGA | 60 |
| KEAP1-Ex-3bR | ACCTTGTGGGCCATGAACTG | |
| KEAP1-Ex-3cF | GCCAGCAGCTGTGTGACGTC | 60 |
| KEAP1-Ex-3cR | ACTTCTCGCCCATGGAGATG | |
| KEAP1-Ex-3dF | CCAAGGTCATGGAGCGCCTC | 60 |
| KEAP1-Ex-3dR | TGGTCCTTCTCCTGACACTG | |
| KEAP1-Ex-4aF | GTGACTGGAGAGTCAGCCCG | 60 |
| KEAP1-Ex-4aR | ACTTCTGCAGCTGCATCTGC | |
| KEAP1-Ex-4bF | CATCAACTGGGTCAAGTACG | 56 |
| KEAP1-Ex-4bR | GGTTGTAAGCCTCCAGGTAG | |
| KEAP1-Ex-4cF | AGGTGGGCCGCCTGATCTAC | 56 |
| KEAP1-Ex-4cR | GCGACCACTGATTGGTCATG | |
| KEAP1-Ex-4dF | AACTCGCCCGACGGCAACAC | 54 |
| KEAP1-Ex-4dR | GACTTGCCAGGAGCAGGACC | |
| KEAP1-Ex-5F | GTCAGCTATAATGGCCATTG | 60 |
| KEAP1-Ex-5R | TGTTCCTGGGTGCTCCCCTC | |
| KEAP1-Ex-6F | TCCCAAAGCCAGACCCCCAG | 60 |
| KEAP1-Ex-6R | AGATGGGCTAGTCAGGACTC | |
| KEAP1-Ex-7aF | TCTTGGATGTGGTGTGACAG | 56 |
| KEAP1-Ex-7aR | TGATACTCCCCATTGGACTG | |
| NFE2L2- Ex2-F | CCACCATCAACAGTGGCATA | 64 |
| NFE2L2-Ex2-R | CCTGCCATAACTTTCCCAAG | |
| **Gene expression** | | |
| KEAP1 taqman assay | Hs00202227_m1* | |
| HuPO taqman assay | 4326314E | |
| **QMSP** | | |
| KEAP1-meth_forw | TGCGGTCGTCGGATTACGAGGTCG | |
| KEAP1-meth_rev | CTTCCATCTCCCGATTTCGTTAC | 66 |
| KEAP1-meth_probe | FAM-GTGGCGCGTAGTTTCGCGAG-TAMRA | |
| ACTB-forw | TGGTGATGGAGGAGGTTTAGTAAGT | |
| ACTB-rev | AACCAATAAAACCTACTCCTCCCTTAA | 55 |
| ACTB-probe | FAM-ACCACCACCCAACACACAATAACAAACACA-TAMRA | |

*Taqman gene expression assay from Life Technologies, Thermo Fisher Inc.

**Supplementary Table 2: Clinical, tumor stages and histological features of cRCC affected patients from the TCGA data Portal ($n$ = 481)**

| Characteristics | *n* (%) |
|---|---|
| **Age (yy ± SD)** | 62 (26.5–90) |
| Sex | |
| M | 314 (65) |
| F | 167 (35) |
| **Tumour Stage** | |
| 1 | 214 (45) |
| 2 | 47 (10) |
| 3 | 127 (26) |
| 4 | 93 (19) |
| **Tumor Dimension** | |
| 1 | 220 (46) |
| 2 | 65 (13) |
| 3 | 181 (38) |
| 4 | 15 (3) |
| **Lymph nodes** | |
| N0 | 204 (94) |
| N1 | 14 (6) |
| Missing | 263 |
| Metastasis | |
| M0 | 364 (81) |
| M1 | 88 (19) |
| Missing | 29 |
| **Fuhrman Grading** | |
| Grade I | 8 (2) |
| Grade II | 196 (41) |
| Grade III | 189 (39) |
| Grade IV | 83 (18) |
| Missing | 5 |
| **OS** | |
| Death | 164 (34) |
| Alive | 317 (66) |

Data are reported as mean (IQR) for continuous variables and as frequencies and percentages for categorical variables.; OS, Overall Survival.

**Supplementary Table 3: Renal Cell Carcinoma tumor tissues evaluated for the presence of *KEAP1* and *NFE2L2* genes alterations**

| ID Patients | Histology | *KEAP1/NFE2L2* alterations | *KEAP1* Methylation* |
|---|---|---|---|
| RCC-010 | ccRCC | absent | no |
| RCC-023 | ccRCC | absent | yes |
| RCC-024 | ccRCC | absent | yes |
| RCC-028 | ccRCC | absent | no |
| RCC-097 | ccRCC | absent | no |
| RCC-098 | ccRCC | absent | yes |
| RCC-099 | ccRCC | absent | yes |
| RCC-101 | ccRCC | absent | yes |
| RCC-128 | ccRCC | absent | yes |
| RCC-129 | ccRCC | absent | no |
| RCC-131 | ccRCC | absent | no |
| RCC-134 | ccRCC | absent | no |
| RCC-137 | ccRCC | absent | no |
| RCC-140 | ccRCC | absent | yes |
| RCC-141 | ccRCC | absent | no |
| RCC-142 | ccRCC | absent | yes |
| RCC-143 | ccRCC | absent | yes |
| RCC-145 | ccRCC | absent | yes |
| RCC-146 | ccRCC | absent | no |
| RCC-147 | ccRCC | absent | yes |
| RCC-058 | PRCC2 | absent | - |
| RCC-060 | PRCC2 | absent | - |
| RCC-061 | PRCC2 | absent | - |
| RCC-062 | PRCC2 | absent | - |
| RCC-084 | PRCC2 | absent | - |
| RCC-085 | PRCC2 | absent | - |
| RCC-086 | PRCC2 | absent | - |
| RCC-087 | PRCC2 | absent | - |
| RCC-088 | PRCC2 | absent | - |
| RCC-089 | PRCC2 | absent | - |

*Methylation data were reported only for the Clear Cell histotype, where a statistically significant difference was found between paired normal and tumour tissues. ccRCC, clear cell Renal Cell Carcinoma. PRCC2, Papillary Tipe 2 Renal Cell Carcinoma.
